# Disaster management training for environmental health: A narrative literature review

**DOI:** 10.4102/jamba.v16i1.1706

**Published:** 2024-09-30

**Authors:** Patience Mbola, Davies V. Nkosi, Oyewale M. Morakinyo

**Affiliations:** 1 Department of Environmental Health, Faculty of Science, Tshwane University of Technology, Pretoria, South Africa; 2 Department of Environmental and Occupational Studies, Faculty of Applied Science, Cape Peninsula University of Technology, Cape Town, South Africa; 3 Department of Environmental Health Sciences, Faculty of Public Health, University of Ibadan, Ibadan, Nigeria

**Keywords:** environmental health practitioners, disasters, disaster management, health emergency, training, education, capacity building

## Abstract

The growing frequency and severity of disasters worldwide have highlighted the need for environmental health practitioners to be equipped with specialised training to respond effectively to evolving public health contexts. Disasters can have long-lasting impacts on the environment and environmental health services, necessitating prompt and effective responses. However, the current environmental health workforce faces challenges in acquiring the necessary competencies to address environmental health threats during disasters. This narrative review synthesises existing literature on disaster management education for environmental health professionals, exploring current training, advancements and emerging trends. The review follows Preferred Reporting Items for Systematic Reviews and Meta-analyses guidelines and includes a total of 45 records that met inclusion criteria (compromising 15 peer-reviewed articles and 30 training records) published between 2002 and 2023. Findings highlight the expansion of environmental health degree programmes to include disaster management, better preparing newly qualified practitioners. However, a knowledge gap remains for previously qualified practitioners. High-income countries prioritising capacity building for environmental health practitioners in disaster management are better equipped to respond to and mitigate disasters.

**Contribution:**

The review suggests that with proper basic training for disaster responders, more lives can be saved during and after disasters. It highlights the insufficiency of current training programmes and emphasises the need for advanced role-specific training for environmental health practitioners. The review emphasises the need for advanced role-specific training, community assessment skills and focused disaster response strategies to enhance environmental health practitioners’ ability to respond to disasters and improve public health resilience. Enhanced training, capacity building and collaboration are necessary to improve the competencies, skills and knowledge of environmental health practitioners in disaster risk management and public health emergencies.

## Introduction

With the global increase of disasters resulting from natural hazards (such as floods, droughts and heatwaves) and human-induced hazards (e.g. landslides, industrial accidents like chemical spills, etc.), the world is in constant need to prepare; respond and reduce public health impacts. Between 2000 and 2019, the world recorded at least 7348 disasters associated with natural hazards that resulted in about 1.2 million deaths and affected over 4 billion people (Centre for Research on the Epidemiology of Disasters 2020; Hung et al. 2021). These disasters usually occur when they are least expected and health systems are unprepared (Gonzale, Souris &Valdivia 2018). In addition, such disasters have a likelihood of prolonged impact on the environment and the environmental health (EH) services (such as water supply, wastewater disposal, solid-waste handling, air and soil quality, food hygiene, vector control and overcrowding) of the affected areas (Eldridge & Tenkate 2018; Généreux, Lafontaine & Eykelbosh 2019; South Africa: Department of Health 2013). In line with the requirements, environmental health practitioners (EHPs) are required to have been registered (licensed) into a professional board such as the Health Professions Council of South Africa (HPCSA) as independent practitioners before practicing (South Africa: *Health Professions Act 56* of 1974). Thus, EHPs are essentially responsible for environmental surveillance, hazardous materials management, vector control and ensuring food and water quality (South Africa: Department of Health 2013). Noteworthy is the fact that while EHPs work within communities, they also cooperate with food companies, research institutions and governments during emergency response strategy development. Whereas EHPs’ participation in responding to disasters follows the same principles applied to their routine professional practice, for them to work effectively, they need to be acquainted with the emergency management systems. Hence, they must be equipped with the necessary skills and training to work in a disaster setting and be able to work cooperatively with other government and non-government agencies (Gerding et al. 2020; Murphy 2020).

Even though EHPs have been delegated with the stated critical responsibilities to prevent health impacts during disasters, globally, the EH workforce continues to grapple with insufficient competencies that inhibit them from responding to public health threats timely and properly (Mbazima, Mbonane & Masekameni 2022; Ning et al. 2014; Kaba et al. 2020; Reischl & Sarigiannis 2008; Shah 2022). The increase in demand for well-trained public health professionals who can convey and address the changing context of global health challenges in communities concerning disasters emphasises the urgent need for EHPs to be trained in disaster responses and management (Miles 2016; Shah 2022). In addition, the Sendai Framework for Disaster Risk Reduction (SDFF) has also emphasised the importance and need of enhancing the resilience of health systems through training and capacity development of health professionals and integrating disaster management into all spheres of healthcare (Ryan et al. 2013; Sendai Framework for Disaster Risk Reduction 2015).

**FIGURE 1:**
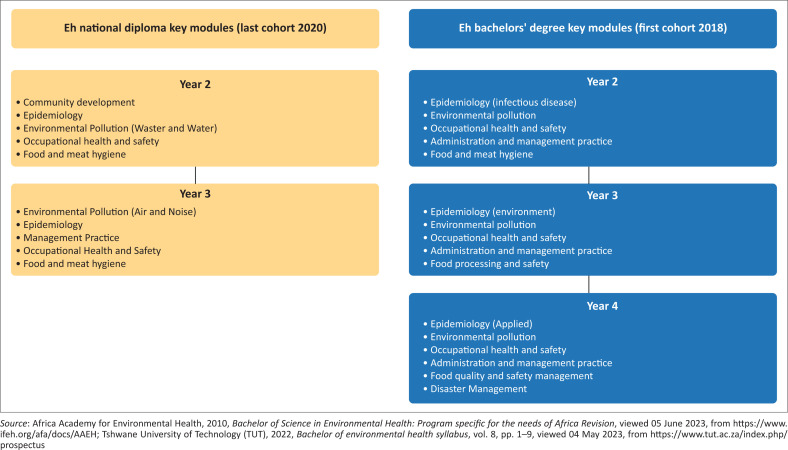
Changes in the environmental health curriculum from the national diploma to the bachelor’s degree and the inclusion of disaster management.

Evidently, including disaster management as a standalone module in the EH curriculum was a significant contribution to the field (Kalis & Zaidel 2016 & Patthanaissaranukool, Fongsatitkul & Warodomrungsimun 2020; Ryan et al. 2013). Based on this view, it was evident that the recent curriculum review recognised the importance of having an inclusive curriculum that addresses the ever-evolving environmental health challenges. Significantly, these reviews were in line with the Sendai Framework (SFDRR), which emphasises the need for human health-centred disaster risk management and emphasises the importance of strengthening disaster risk capacities to protect communities (SDFRR 2015). The importance of disaster management training or capacity building of EHPs training at the university level was then advocated and given their historical role as a support structure in emergencies such as drought, wildfires, hurricanes, infectious diseases outbreaks, wars and unrests, floods and droughts. Though rightfully included, the challenge was that the inclusion of disaster management training in EH was not done in the past, especially in low-income, lower middle-income and upper middle-income countries (Kaba et al. 2020; Patthanaissaranukool et al. 2020; Perpiñá-Galvañ et al. 2021; Ning et al. 2014; Shah 2022). To respond to this new concept, the African countries represented by the Africa Academy for Environmental Health (AAEH 2010) resolved to harmonise their curriculum focusing on current disaster risk responses and disaster understanding by communities, especially in the 21st century (African Academy for Environmental Health 2010; Jepngetich et al. 2018; Shah 2022). With all these changes low-income, lower middle-income and upper middle-income countries were not left behind (AAEH 2010; Department of Higher Education and Training 2012; Jepngetich et al. 2018; Patthanaissaranukool et al. 2020; Shah 2022; Tshwane University of Technology [TUT] 2022). For instance, in the case of South Africa, the EH degree is offered by five universities of technology and two traditional universities at a National Qualification Framework (NQF) level 8, with disaster risk management included in the 4th year level (see Figure 1) (AAEH 2010; DHET 2012; TUT 2022). Furthermore, the EH degree focuses on equipping students with a thorough grounding in the knowledge, theory, principles and skills of the profession and the ability to apply these to the EH career context through work-integrated learning (DHET 2012). The packaging of disaster management was seen as a great contribution as EHPs are generally playing a proactive role in different communities. The training of these candidates in the new 4-year degree programme signalled a positive inclusion. Subsequently, the 1st cohort of students trained in this new programme was in 2018 and started to practice as qualified EHPs in 2019 and were registered with the compliance body (Cele et al. 2012). It is important to note that EH has evolved over the years. Figure 1 shows the different changes in the EH curriculum from when it was offered as a National Diploma until when disaster management was introduced as a module in the EH degree (DHET 2012; South Africa: *Health Professions Act 56* of 1974).

Literature shows that EHPs have long been involved in the control of disasters (during the control of disease outbreaks such as the bubonic plague, smallpox outbreaks, listeriosis and most recently coronavirus disease 2019 [COVID-19]) (Mäki 2008; Rodrigues et al. 2020; World Health Organization 2018). The role of EHPs has always been emphasised in different modules (such as epidemiology, food processing and safety etc.) and not necessarily disaster management; hence the module evolution had not been given much attention in EHPs’ training curriculum (Eldridge & Tenkate 2008; Gochfeld 1999; Health Professions Council of South Africa 2019; Mäki 2008). While certain aspects of dealing with issues such as the emergence of outbreaks in modules were covered in the diploma, it was not sufficient for handling and participating in disaster response for EHPs. The inclusion of the disaster management module in the new degree will put the newly qualified EHPs in a better position to handle emerging disasters while the need remains for the EHPs who qualified under the diploma to receive such training to purge the knowledge gap. Consequently, a disaster-management curriculum that is designed to train future and current EHPs, to prepare, respond and recover from the adverse impact of disaster hazards by the accredited higher institution of learning, professional bodies and other organisations remains a necessity (Ryan et al. 2013). This systematic review looks at current environmental health disaster management training trends, their purposes and packaging for the new EHP.

## Research methods and design

**FIGURE 2:**
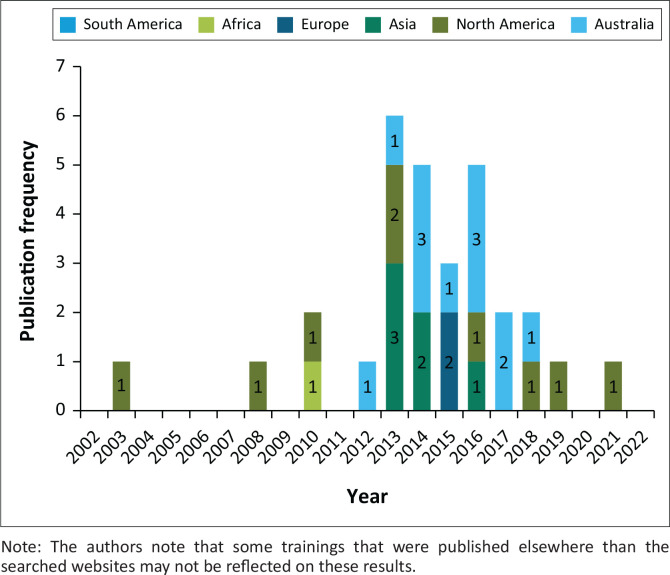
Disaster management training in the environmental health profession conducted between 2002 and 2023.

This review adopted a narrative review to explore and synthesise the existing literature on disaster management education for environmental health professionals highlighting current training, advancements and trends as well as identifying knowledge gaps. The narrative review is designed to summarise and describe findings of several studies (key literature) to identify themes patterns and trends; hence this method was adopted for this study (Green, Johnson & Adams 2006). To ensure transparency and rigour, this research adheres to the Preferred Reporting Items for Systematic Reviews and Meta Analyses guidelines (PRISMA) guidelines, which provide a standardised framework for reporting systematic reviews (Ferrari 2015; Moher et al. 2009). A systematic search for the relevant literature was conducted, which incorporates peer-reviewed and published studies providing insights into the contents of disaster management training for the EH profession. To support this view, grey^1^ material from different web pages of EH was searched for updated information reported on EH disaster management trainings conducted during the investigation periods (shown in Figure 2).

### Inclusion criteria

**FIGURE 3:**
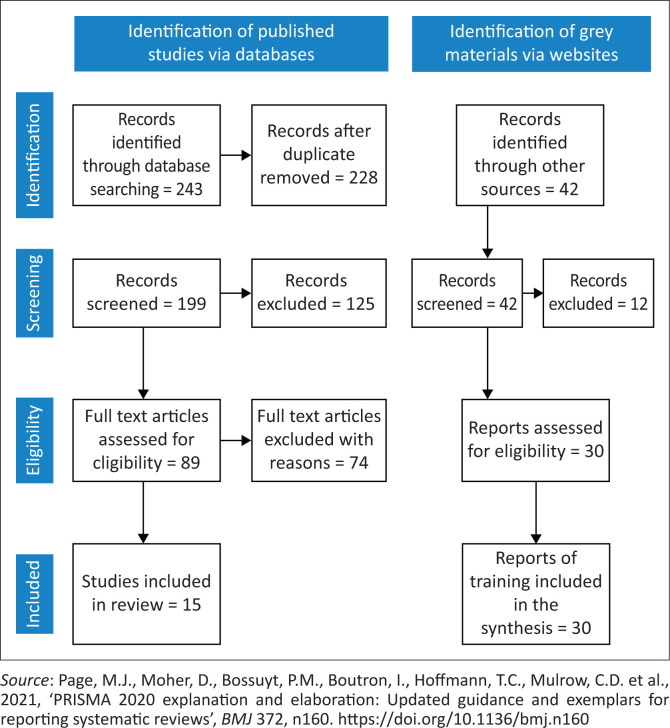
PRISMA flow diagram of the search process.

The search strategy for this review involved using the key terms: ‘health emergency and education OR training AND disaster management AND environmental health in combination’. The search strategy included a comprehensive search of three electronic databases (Google Scholar, ProQuest and Science Direct) as well as a targeted search of relevant websites, such as Institutes, of Environmental Health and International Federation of Environmental Health (IFEH) for English-published articles and grey material, which included documentation and records of disaster training for EHPs. The search covered the period from 2002 to 2023. Only full-text articles that were available to the authors were considered from the databases. Articles resulting from these databases and grey materials from websites were then exported; duplicated records were then removed as per PRISMA requirements. Figure 3 presents a search strategy and flow diagram output as presented in the article.

### Exclusion criteria

Published articles and training programmes were excluded if they had no relevance to EH disaster management training. Postgraduate theses and papers written in languages other than English were also excluded. The results of the disaster management for EH, focusing on the country of origin, research type and recommendations, are presented in a figure (Figure 2) representing training conducted and a table (Table 1) reporting on peer-reviewed articles published.

**TABLE 1:** A list of published peer-reviewed research articles conducted on environmental health disaster management training, competencies and skills (2002–2023).

Country	Aim	Research type	Study findings and recommendations	Sources
Australia	To review accredited formal qualifications for DRR and CCA capacity development	Concept paper	- International Federation of Environmental Health has set an example of DRR and CCA accreditation by providing short course EH DRR training recognition. - Training is delivered by a combination of partner universities (Griffith) and EHPs across the International Federation of Environmental Health Asia and Pacific Region.	Hemstock et al. (2016)
California	To explore the capacity of EH and EPR programmes to facilitate participatory relationship between themselves, and with the community members, they serve and to assess past levels of community emergency preparedness outreach	Research article	- Ambiguity still exists pertaining to the EH functions in disasters and poor representation of EHPs in disaster planning. - Little attention has been paid to the EHPs’ training needs for responding to bioterrorism and other public health emergencies. - Critical need for the development of effective training on community-based participatory methodologies that would prepare EHPs to engage communities by building partnerships for disaster resilience capacity.	Gamboa-Maldonado et al. (2012)
Caribbean (Barbados, Guyana, Jamaica, Saint Lucia, Trinidad, and Tobago)	To assess the effectiveness of training received by the EH inspectors	Research article	- EHPs to be competent in disaster risk management and mitigation. - Increasing need for universities to strengthen disaster risk management approaches in the curriculum. - Poor human resource development and systems serves impact EHPs’ preparedness to meet increasing responsibilities. - Policymakers and educators need to reassess public health systems, performance and regional investment improvements.	Shah (2022)
China	To determine the emergency preparedness competency specific to PHIs, preparedness limitations and needs of the workforce, as well as to identify important factors that affect the preparedness competency of PHIs	Research article	- Unsatisfactory performance in knowledge and skills among PHIs regarding emergency preparedness. - An improvement of the relevant knowledge and skills for PHIs should be prioritised in public health emergencies. - Ambiguity in emergency responsibilities is the most important factor undermining the preparedness competency of PHIs.	Ning et al. (2014)
Ethiopia	This research paper aimed to assess the perceived knowledge, experiences and training needs of health professionals (including EHPs) regarding disasters, their prevention and management in Jimma Zone, Southwest Ethiopia	Research article	- A considerable number of health professionals in this study had limited knowledge about the concept of disaster and response to certain specific disasters. - This study findings indicated that most health professionals lacked opportunities to update themselves on the latest and broader perspective of disaster prevention and disaster management. - The study recommended well-organised programmes designed to provide refresher training for health professionals at a desirable interval.	Berhanu et al. (2016)
Kenya	To assess the graduates’ preparedness to handle emerging public health concerns	Research article	- Training content seemed to be below the demand of the job. - Participants felt inadequately prepared to handle emerging public health challenges. - The majority of the EH graduates (participants) expressed willingness to participate in public health emergencies and therefore called for adequate and proper training. - There is a need of collaboration between the higher education management and field practitioners to: ◦ Review curriculum content and adapt it, to meet educational approaches and enhance competency scores.	Jepngetich et al. (2018)
South Africa	To evaluate the state of EH in South Africa	Review investigation	- Knowledge gaps and inconsistencies regarding the investigation of communicable disease. - Training of EHPs should be frequently reviewed and adapted with the current EH challenges.	Mbazima et al. (2022)
Uganda	To assess the professional competencies and training needs of EHPs in Uganda	Cross-sectional survey research article	- Disaster preparedness and infection prevention control competencies were identified to be lacking amongst EHPs in Uganda. - Emerging and re-emerging disease outbreak preparedness and disaster management should be strengthened in the EH curriculum for undergraduate students and included in the continuous development of the field EHPs.	Walekhwa, Kizza & Musoke (2022)
United States of America	To outline the public health lessons learned from Hurricane Katrina and to look at how public health providers can better prepare for disaster response in the future	Editorial article	- Much training on public health preparedness since the September 11 terrorist disaster, nonetheless the effectiveness of this training after the Hurricane Katrina disaster was questionable. - The need was identified to re-examine the effectiveness of disaster preparedness training for public health practitioners. - Public health professionals need to reflect on the progress of previous disasters and to improve the level of response.	Logue (2006)
United States of America	Identifying training gaps and emerging issues in EH disaster preparedness	Journal article	- EHPs’ involvement in disaster process includes responsibilities such as community assessment, informing re-entry decisions in evacuated areas. - Maintaining awareness of primary and secondary hazards and vulnerable populations are important for effective self-preparedness of EHPs and community and individual resilience.	Rubin, Walker and Allan (2021)
United States of America	Identification of future research needs in the EH field 15 years after Hurricane Katrina	Editorial article	- Post Hurricane Katrina EH actions focused on worker exposure and community needs assessments. - Future research should focus on strengthening and measuring community resilience. - Communities’ EH literacy may result in the protection of the most vulnerable from disasters.	Litchveld and Birnbaum (2020)
United States of America	Providing opportunities of continuing education and professional growth in the field on environmental health to NEHA members	Editorial article	- NEHA contributed in capacity development in emergency response for EHPs, by providing opportunities for professional growth (since 2007). - EHPs can either participate in full 5-day EHTER/the condensed courses/attend the online version.	Collins (2013)
United States of America	To assess the emergency response training needs of the local environmental health in Michigan state	Research Article	- Identified high priority needs of training for EHPs regarding their role in disasters.	Reischl, Sarigiannis and Tilden (2008)
United States of America	To identify perceived preparedness and response training needs for the CDC responder workforce	Research article	- There is a need for more advanced, role-specific training, and experiential opportunities to reinforce concepts and practice. - Exercises are a highly effective approach to training responders. - Regular needs assessments should be conducted to expand training and remain responsive to the complexities of emerging threats.	O’Meara et al. (2019)
United States of America, United Kingdom, Canada and Australia	To review the job description of EHP and qualifications to become a Registered EH Specialist/Registered Sanitarian in selected countries	Review paper	- EHPs in the USA are expected to complete the NEHA examination with core competencies related to the EH field. - Disaster management forms 3% of this examination. - USA reflected on contributing to the skills and competencies of EHPs regarding response to emergencies.	Patthanaissaranukool et al. (2020)

CCA, climate change adaptation; CDC, center for disease control and prevention; DRR, disaster risk reduction; EH, environmental health; EHP, environmental health practitioners; EPR, emergency preparedness and response; EHTER, environmental health training in emergency response; NEHA, national environmental health association; PHIs, public health inspectors.

Note: Please see the full reference list of the article, Mbola, P., Nkosi, D.V. & Morakinyo, O.M., 2024, ‘Disaster management training for environmental health: A narrative literature review’, *Jàmbá: Journal of Disaster Risk Studies* 16(1), a1706. https://doi.org/10.4102/jamba.v16i1.1706, for more information.

### Ethical considerations

Ethical clearance to conduct this study was obtained from the Tshwane University of Technology, Research Ethics Committee (No. REC2023-12-087).

## Results

Despite the scarcity of information on EHPs’ training in disaster management, our search yielded a significant amount of relevant data. When looking at continental contributions, we found 30 disaster training records represented as ‘grey material’ (Figure 2) and 15 published peer-reviewed papers (Table 1) that met the inclusion criteria. Majority of the disaster management trainings in EH have been found to be from Australia 12 followed by North American countries nine (9). Six were from Asian countries while only two were from European countries. Only one training relating to disaster management was found in African countries, which focused on the generic curriculum for EH degrees in Africa. No disaster management training for EH was found in South America and Antarctica from the website search between 2002 and 2023. Most of the training recorded from the websites only started after 2008 for all the continents, with more training conducted between 2013 and 2016. Given the number, high-income countries were seen as better responding to the emerging needs of equipping EHPs with skills for preparation and response to disasters. Most of the identified training courses (published on the searched websites) participants were on-the-job EHPs (currently working EHPs) and the duration of the training lasted between 2 and 6 days. The most common focus of these trainings was on the role of the EH in disaster management, mitigation of environmental health risks during disasters and guiding principles to assess, address and respond to environmental health impacts during disasters. Common hazards based on local contexts such as floods, hurricanes, hazardous chemical spillages, tornadoes and cyclones have been utilised to provide case studies for the training of the EHPs.

Table 1 provides a summary of published peer-reviewed research articles conducted on EH disaster management training outcomes and recommendations. A total of fifteen (15) papers searched globally obtained from Google Scholar ProQuest databases met the inclusion criteria for the search period between 2002 and 2023. The results indicate that high-income countries had more programmes dedicated to training and increasing the capacity of EHPs regarding disaster management. The consensus is that there is an increased demand for focused training for EHPs on disaster management, emergency response, coordination and collaboration during a disaster.

## Discussion

This review gives us an overview of the need for continuous professional development in disaster risk management among the EHPs. Given the increasing challenges confronting the EH workforce to keep up with acquiring skills that may enable them to deal with continuously emerging disasters and state of emergencies, the EH curriculum review that considered the inclusion of disaster management as the stand-alone subject came at the right time (see Figure 1). The development of the environmental health curriculum for the new degree has been shaped by the guidelines of the Association of African Environmental Health (AAEH), which seek to enhance environmental health training and service delivery, including emerging areas like disaster management. Moreover, the curriculum is underpinned by key legislation, including the *Health Professions Act*, 1974 (Act No. 56 of 1974), which defines the scope of practice for environmental health professionals (South Africa: *Health Professions Act 56* of 1974); National Health Act 2003 (Act No.61 of 2003), which sets environmental health norms and standards (South Africa: National Health Act 61 of 2003), and the (National Environmental Health Policy 2013) which prioritises the prevention of harmful environmental and health behaviours, all of which collectively shape the desired role of EHPs (South Africa: Department of Health 2013).

Nonetheless, while the current EH degree students will possess an understanding to assess, address and respond to the EH impact that may be caused by disasters, there remains a critical need for bridging a competency and/or skills gap, especially among the EHPs that qualified with a Diploma where the subject of disaster management subject was not included (Berhanu et al. 2016; Jepngetich et al. 2018; Ning et al. 2014). The bridging of this gap must be informed by ever-changing contexts and by regular training needs assessment to remain responsive to emerging threats and challenges. Training of past and future EHPs is expected to be part of a disaster response strategy and remains important in saving lives (Berhanu et al. 2016; Gerding et al. 2020; Mbazima et al. 2022; Ning et al. 2014). Furthermore, while disaster management skills are essential, particularly for EHPs who qualified before the introduction of disaster management modules, the environmental health scope of practice should also acknowledge and address these skill gaps (Walekhwa et al. 2022). Moreover, the scope of practice should integrate continuous professional development requirements that focus on building the capacity of EHPs in disaster response, ensuring they are equipped to effectively manage disasters and emergencies, especially the development of Short Learning Programmes tailor made for this intervention strategy (HPCSA 2021).

As shown in Figure 2, high-income countries had more training published for their EHPs aimed at equipping them for disaster response than middle-income and low-income countries, these included on-programme training (for undergraduate EH students) and on-the-job training (for practicing EHPs). It was surprising to note that African countries had no training published for building the capacity of EHPs to prepare them for disasters, especially as the continent is vulnerable to many disasters attributable to natural hazards such as floods/droughts and heatwaves (Centre for Research on the Epidemiology of Disasters 2020). Even though many high-income countries had more recorded training for their EHPs, published research articles (see Table 1) still point out the shortage of continuous training in disaster management for EHPs worldwide (Litchveld & Birnbaum 2020 & Shah 2022; Logue 2006; O’Meara 2019). Some researchers have associated the lack of EHPs’ preparedness to handle disasters with their low representation in the disaster management phases and the ambiguity of their responsibilities in emergencies (Dhesi 2018; Gerding et al. 2019; Rodrigues et al. 2021). Other researchers further suggested that the training of EHPs will further result in their role clarity in emergencies and disasters (Eldridge & Tenkate 2008; Ryan et al. 2013; Ning et al. 2014). It is expected that with proper basic training of proactive disaster responders, more lives could be saved during disasters and emergency responses (Hung et al. 2021; Perpiñá-Galvañ et al. 2021; Ryan et al. 2013). Furthermore, as seen from this review very few published research papers, only 15 papers in the period of 20 years (see Table 1) focusing on the capacity and training or improvement of the relevant knowledge and skills of EHPs to prepare for emergencies and disaster events, this suggests a scarcity of research in this area.

Evidently, the role of EHPs cut across all the stages of disaster management and intervention strategies must include hazard identification as part of proper vulnerability assessment, informed disaster risk response strategies and community training and awareness (Rodrigues et al. 2021; Ryan et al. 2017). Therefore, professional development courses that are aimed at equipping the EHPs who qualified before the introduction of disaster management in the curriculum should be designed to train EHPs on strengthening and measuring community resilience (Gamboa-Maldonado et al. 2012; Litchveld 2020; Mbazima et al. 2022; O’Meara et al. 2019; Reischl et al. 2008).

### Training environmental health practitioners on community assessment to enhance disaster resilience

**FIGURE 4:**
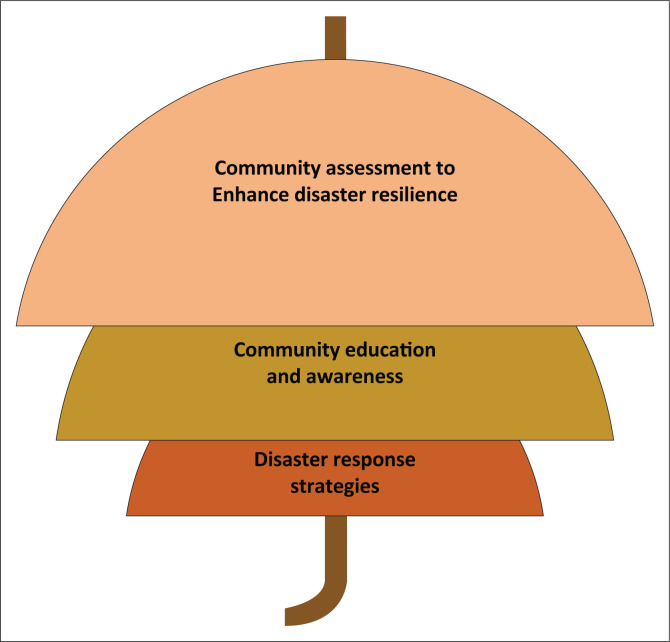
Environmental health training framework for community shielding against disasters.

Environmental health practitioners’ interventions during disasters include the responsibility of reducing the vulnerability of communities to hazards and increasing their capacity to respond, withstand disruptions and recover rapidly (Abrash-Walton 2021; Ryan et al. 2017). Moreover, maintaining awareness of primary and secondary hazards and vulnerable populations is important for the effective self-preparedness of EHPs and for community resilience (Rubin et al. 2021). In addition, identifying vulnerable populations is central to the goal of strengthening the overall resilience of a community (Woods, Sabogal & Kalis 2022). Hence EHPs need to be trained in skills linked with community assessment before a disaster. This should include vulnerabilities to different hazards for each community, thus developing specific disaster response strategies in an event of eventuality (Gamboa-Maldonado et al. 2012; Miranda et al. 2021; Ryan et al. 2013; eds. Wisner & Adams 2002). This training will not only give EHPs skills to estimate disaster impacts but also preparation strategies for each community. This is noted by Wisner and Adams (eds. 2002), asserting that vulnerability assessment training will enable the EHPs to predict and map the risks that a community will face in the event of a disaster and during the recovery period. A study by Shah (2022) recommended the strengthening of disaster risk-management approaches (vulnerability assessment) in EH to enhance the understanding and skills of EHPs. Environmental health practitioners who are sufficiently trained to anticipate the problems that may emanate from disaster will be in a better position to reduce the vulnerability of communities to hazards and thus increase their ability to respond and withstand disaster disruptions through devising response strategies (Gamboa-Maldonado et al. 2012; Rodrigues et al. 2021). Figure 4 shows the assessment of communities’ vulnerabilities as a major shield to minimise disaster impacts. This will enable proper resource allocation and identification of response plans.

### Community education and awareness

Community education and disaster awareness is the second shield to be incorporated into EH training. While in general EHPs are trainers, their ability to pass messages and information in different communities cannot be over-emphasised (Woods et al. 2022). Wisner and Adams (eds. 2002) noted that public awareness creation and mobilisation programmes by EHPs are necessary in reducing disaster vulnerability, by informing the community how disaster impact can be reduced; increasing community awareness and thus encouraging people to protect themselves and their environment from a disaster. Environmental health practitioners are not only expected to impart community education during disaster, but it is also part of their routine activity forming part of their role. Ryan et al. (2013) argued that communication is one of the important tools to promote self-sufficiency of the community during disaster.

### Disaster response plans and strategies

Informed disaster response plans will facilitate the rapid tackling of mitigation plans and is the third shield that EHPs must be capacitated with. Disaster responses influenced and planned with communities (to increase their resilience) will ensure the timely classification of much-needed plans for survival. Responses must be influenced by the type of hazard and its impact (eds. Wisner & Adams 2002). Part of the response strategy may include the identification of stakeholders, proper coordination and resource allocation (Sun, Bocchini & Davison 2020). Corresponding to an investigation conducted by Ryan et al. (2017), the impact of different hazards is generally minimised by proper and timely responses. For example, disaster responders in Queensland lacked risk assessment tools to assist them in disaster preparation for response. This resulted in limited information being collected for decision makers to determine priority areas promptly and allow public health interventions to be based on evidence (Ryan et al. 2013). Numerous studies have shown that lack of disaster knowledge among EHPs resulted in professionals feeling inadequately prepared to handle public health challenges (Jepngtich et al. 2018; Ning et al. 2014; Shah 2022).

### Limitations

Several limitations exist in this study. Initially, the search for the published or documented disaster training for EHPs was restricted to those published on the IFEH website and those reported on the Google search engine. This may have excluded relevant training published elsewhere or not published at all. Additionally, research articles published in databases other than Google Scholar, Science Direct and ProQuest may not have been included. Lastly, the study only considered articles and documentation published between 2002 and 2023, and any relevant information published after this period was not taken into consideration. Therefore, the results of this study regarding the capacitation and training of EHPs in disaster management cannot be fully generalised in the global context but to low-income to middle-income countries.

## Conclusion

The impact of climate change and the increasing intensity of disaster events will continue to necessitate adequately trained EHPs to control environmental factors with the potential to affect public health. The review indicated that the inclusion of disaster management in the EH curriculum will produce EHPs with readiness to contribute to the related disaster response needs with a danger to cascade health emergency. In addition, the findings pointed to more areas that must be strengthened in the EH disaster management training curriculum, to enhance the understanding of the EHPs. Community resilience is one of the key areas to which attention should be given as far as training is concerned as they play a proactive role in reducing vulnerabilities of community to disasters. Prioritising training will assist EHPs in clarifying their roles in disasters, giving more attention to vulnerability assessment, which will result in enhancing community resilience in responding to disasters, disaster response plans and community awareness and education will yield community safety during disasters. Environmental health practitioners need to be trained to the extent that they can conduct assessments and create awareness regarding the anticipated disaster risks to the populations. Therefore, short courses that are aimed at equipping EHPs who qualified before the inclusion of disaster education in the EH curriculum need to be prioritised to purge the existing gap and in response to the new challenges. Consequently, the collaboration between higher education and field EHPs is essential to review and adapt the curriculum content to meet the current demands of the EHPs. This paper highlights the need for enhanced training, capacity building and collaboration to improve EHPs competencies in disaster management and environmental health challenges.

## Footnotes

1 Grey material: This is published advertisements for training, legislated documents, patent rights and other website advertising EH training information other than published peer reviewed articles.
